# Depression is a core symptom of multiple sclerosis: Commentary

**DOI:** 10.1177/13524585241245637

**Published:** 2024-04-30

**Authors:** Anthony Feinstein

**Affiliations:** Sunnybrook Health Sciences Centre, University of Toronto, Toronto, ON, Canada

The Controversies section in this month’s journal addresses a perennially challenging question: Should depression be regarded as a core symptom of multiple sclerosis (MS)? Dr. Leavitt argues that it should not, opining that by doing so, we assign a special status to MS-related depression implying it is biologically distinct from depression occurring in other neurological and psychiatric disorders. Rather, she suggests, understand depression as a comorbid feature of MS.^1^

Dr Hancock,^2^ on the other hand, cites a wealth of epidemiological, neuroimaging, immunological, and treatment data to argue that depression is very much a core symptom of MS, noting that Charcot recognized it as such in his seminal description of the disease.

The fact that we are still debating an observation that is over a century old speaks to the lingering tensions and uncertainties that characterize our understanding of dualism and the mind-body relationship.^3^ Dr. Leavitt correctly observes that depression is non-specific, occurring in many other conditions. But then again, so too is visual disturbance, incontinence, paresthesia, and a host of additional physical difficulties, all of which are readily accepted as symptoms of MS without clinicians challenging their validity as evidence of the disease. Which begs another allied question: Why is depression held to a different standard? This article’s word limit precludes a detailed answer, but a few pertinent observations can help frame why we are still having this discussion in the 21st century.

Depression is synonymous with sadness, a ubiquitous normal emotion when mild and transient. Who has not felt momentarily sad when confronted by one of life’s disappointments? Depression, however, lies along a continuum, from the mildness of an isolated, brief symptom to a more ominous, potentially life-threatening syndrome that comes with other well-defined features like changes in sleep, concentration, suicidal intent, and so forth. People with MS can be found all along this spectrum, but as epidemiological data show, they are more likely to occupy the upper reaches relative to healthy, age- and sex-matched samples.^4^

Advances in neuroscience, particularly brain imaging, are starting to offer explanations for this.^5^ Imaging findings now account for almost 50% of the variance in explaining depression in people with MS, an impressive testimony to strides made in recent research.^6^ However, this still lags behind etiological explanations for nonspecific physical symptoms. With optic neuritis, for example, there is invariably the observable lesion and/or abnormal evoked potentials as corroborative evidence; not so with depression. And to stay with this point, a failed marriage may trigger depression but does not cause blindness. It is important to remember that the influence of psychological factors on mood explains as much variance as the neuroimaging data.^7^

In short, reductionist explanations sit comfortably with physical symptoms but still fall short when explaining mental phenomena. Depression is abstract, no less tangible for being so, but in medicine, many practitioners are more comfortable with what they can see and measure objectively. With depression, we are not there yet, hence the question at the heart of this Controversies section.

As matters currently stand, there is support for both Drs. Leavitt’s and Hancock’s opinion. But the gap between them is narrowing in the latter’s direction. Will it ever close completely? I would like to think so, if the progress made since Charcot’s time is any pointer. Until it does, however, clinicians must remain vigilant in looking out for depression. Evidence reveals room for improvement here.^8^
